# probeCheck – a central resource for evaluating oligonucleotide probe coverage and specificity

**DOI:** 10.1111/j.1462-2920.2008.01706.x

**Published:** 2008-10

**Authors:** Alexander Loy, Roland Arnold, Patrick Tischler, Thomas Rattei, Michael Wagner, Matthias Horn

**Affiliations:** 1 Department of Microbial Ecology, Universität WienAlthanstraße 14, A-1090 Wien, Austria; 2 Department of Genome Oriented Bioinformatics, Technische Universität MünchenAm Forum 1, D-85354 Freising, Germany

## Abstract

The web server probeCheck, freely accessible at http://www.microbial-ecology.net/probecheck, provides a pivotal forum for rapid specificity and coverage evaluations of probes and primers against selected databases of phylogenetic and functional marker genes. Currently, 24 widely used sequence collections including the Ribosomal Database Project (RDP) II, Greengenes, SILVA and the Functional Gene Pipeline/Repository can be queried. For this purpose, probeCheck integrates a new online version of the popular ARB probe match tool with free energy (ΔG) calculations for each perfectly matched and mismatched probe-target hybrid, allowing assessment of the theoretical binding stabilities of oligo-target and non-target hybrids. For each output sequence, the accession number, the GenBank taxonomy and a link to the respective entry at GenBank, EMBL and, if applicable, the query database are displayed. Filtering options allow customizing results on the output page. In addition, probeCheck is linked with probe match tools of RDP II and Greengenes, NCBI blast, the Oligonucleotide Properties Calculator, the two-state folding tool of the DINAMelt server and the rRNA-targeted probe database probeBase. Taken together, these features provide a multifunctional platform with maximal flexibility for the user in the choice of databases and options for the evaluation of published and newly developed probes and primers.

## Introduction

Diagnostic hybridization and PCR assays employing oligonucleotides as probes/primers (subsequently referred to as diagnostic oligos) are routinely applied for identifying microbes of interest and for studying the composition of polymicrobial communities in clinical, biotechnological and environmental specimens. The accuracy and performance of these molecular assays are intimately connected with the physicochemical characteristics of the diagnostic oligos and their discriminatory capacity against non-target sequences. Various tools are available that assist researchers in the *in silico* development of diagnostic oligos for (groups of) genes and microorganisms of interest (e.g. Ashelford *et al*., 2002; Kumar *et al*., 2006). Such oligos ideally have a high coverage for the target group (i.e. a high percentage of sequences in the target group possess a perfectly matching probe binding site) and a high specificity (i.e. no or only a low number of perfectly matching sequences not belonging to the target group are known and all other non-target sequences have many strongly discriminating mismatches). Furthermore, theoretical thermodynamic criteria, such as Gibbs-free energy; for efficient formation of the oligo-target hybrid and for avoiding non-specific binding to non-target genes should also be carefully considered during the design of a new diagnostic oligo (Yilmaz and Noguera, 2004; 2007). However, even the best *in silico* evaluation can currently only provide an estimate of the actual binding and discriminatory performance of a probe and thus empirical evaluation of the optimal experimental conditions by using suitable target and non-target reference sequences/organisms remains the final requirement during the development of effective new probes (Daims *et al*., 2005; Loy and Bodrossy, 2006).

Most experiments do not involve the *de novo* design and empirical testing of new diagnostic oligos, but employ already-published probes and primers. The frequent interest in such diagnostic oligos is, for example, mirrored in the user statistics of the rRNA-targeted oligonucleotide probe database probeBase (Loy *et al*., 2007), which show 265 705 page views in the year 2007. A considerable problem of naïve application of already-published probes is, however, that their originally intended coverage and specificity might no longer hold true, taken in consideration the rapid accumulation of sequences in public repositories. Periodic evaluation of a diagnostic oligo is thus of utmost importance for its reliable application in molecular assays (Lücker *et al*., 2007).

probeCheck provides a freely accessible, central platform for rapid *in silico* specificity and coverage evaluations of diagnostic oligos against the latest sequence collections of selected phylogenetic and functional marker genes. By integrating various existing (online-) tools and databases, probeCheck offers a number of unique features (e.g. an online version of the ARB probe match tool (Ludwig *et al*., 2004), its combination with ΔG calculations and new data filtering options, and the possibility to query the currently largest rRNA sequence database SILVA (Pruesse *et al*., 2007) and should thus be a useful web resource for all microbiologists interested in the detection of genes or microorganisms with oligonucleotide-based assays.

### Features of probeCheck

**Fig. 1:**
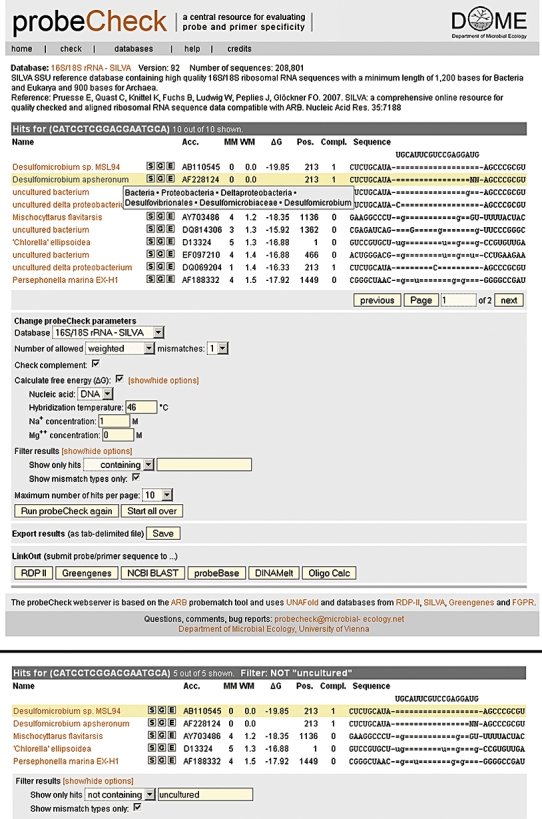
probeCheck screen shot. (Upper panel) An example of the probeCheck output using the *Desulfomicrobium*-specific 16S rRNA-targeted probe DSM213 (probeBase Accession No. pB-00507) (Lücker *et al*., 2007) as query sequence and the 16S/18S rRNA sequence database from SILVA. (Lower panel) Same as above, except that the results were filtered to exclude environmental sequences by excluding hits containing the term ‘uncultured’ in the species/sequence name.

Using a common user interface up to 10 oligonucleotide sequences (8–100 mer, in FASTA format and IUPAC coding, which is resolved automatically) can be queried against a number (currently 24) of sequence databases retrieved from public repositories such as the Ribosomal Database Project (RDP) II (Cole *et al*., 2007), Greengenes (DeSantis *et al*., 2006), SILVA (Pruesse *et al*., 2007) and the Functional Gene Pipeline/Repository (http://fungene.cme.msu.edu/). The probeCheck server employs the established ARB probe match tool, which creates difference alignments of the tested oligonucleotide and complementary sequences as output. The user can adjust the following search parameters. The *check complement* option causes probeCheck to not only search for sequences that are identical to the query sequence (i.e. have the same orientation), but also to target sequences that are in reverse complementary orientation; a feature required for, e.g. checking rRNA-targeted probes used for fluorescence *in situ* hybridization of microorganisms. The *number of allowed weighted* or *unweighted mismatches* can range between 0 and 4. The weighted mismatch value, calculated by the ARB probe match method using default settings, considers the relative strength of base pairings and the position of the mismatch to estimate the stability of the probe-target hybrid. This estimation is best suited for fluorescence-labelled probes applied for whole-cell hybridization (Strunk, 2001), but might also be useful for other hybridization formats such as DNA microarrays (Sanguin *et al*., 2006). In addition, the *free energy (ΔG)* can be determined for each perfectly matched and mismatched oligo-target hybrid by using the two-state hybridization algorithm of the UNAfold software (Markham and Zuker, 2005), allowing rough assessment of the differential theoretical melting properties of oligo-(non-)target hybrids (see Yilmaz and Noguera, 2007 for further information; Loy *et al*., 2005). probeCheck further offers new possibilities for filtering of the results on the output page. A keyword can be entered next to the option *show only hits (not) containing* in order to filter the list of hits for the presence/absence of this keyword in the sequence/species name (see Fig. 1 for an example). Multiple keywords can be entered, separated by ‘OR’. The option *show mismatch types only* restricts the output to only one example sequence per each perfectly matching target and mismatching non-target type, thus presenting a quick overview of the different types of mismatches to the query sequence and facilitating the selection of appropriate non-target references for empirical oligo performance tests.

In the output table (Fig. 1), difference alignments showing the probe binding site and its flanking regions in the target sequence (5′→3′ orientation) are ordered according to the number, position and type of mismatching bases. A short description, the accession number and a link to the respective entry at GenBank (Benson *et al*., 2007), the European Molecular Biology Laboratory (EMBL) database and, if applicable, the query database are given for each output sequence. For rRNA-targeted oligos, the position of the 5′-terminal nucleotide in the oligo binding site relative to the rRNA sequence of *Escherichia coli* is indicated (Brosius *et al*., 1981). The unified NCBI/EMBL taxonomy is displayed on mouse-over the name of each target sequence. The output table can be exported and saved as tab-delimited text file for further processing. In addition, on the results page (Fig. 1) the query sequence can be directly submitted to a number of other web servers for further analysis, including the probe match tools of RDP II and Greengenes, blast (search for short nearly exact matches) at NCBI (Benson *et al*., 2007), the Oligonucleotide Properties Calculator OligoCalc (Kibbe, 2007), the two-state folding tool of the DINAMelt server (enabling evaluation of the oligo's thermodynamic tendency to form self-structures, i.e. hairpins) (Markham and Zuker, 2005) and the rRNA-targeted probe database probeBase (Loy *et al*., 2007).

probeCheck also includes a help page with a detailed description of the input and output features. A separate page contains an overview over the sequence databases available in probeCheck, including information on the release version, a web link to each database homepage, and references.

### Database updates and call for submission of ARB sequence databases

probeCheck is maintained by the Department of Microbial Ecology at the University of Vienna. Databases behind probeCheck are retrieved on a regular basis from public database projects such as RDP II, greengenes, SILVA and FUNGENE, and, if required, are adapted to the ARB database format. The dates of last and upcoming updates are indicated.

Curators of own nucleic acid sequence ARB databases are strongly encouraged to make their databases available for probe/primer evaluations on the probeCheck server. Note that probeCheck only enables matches against the database. The actual database remains hidden in the background and is not available for download. The probeCheck staff can be contacted by email (probecheck@microbial-ecology.net) for questions and bug reports.

### Technology behind probeCheck

The probeCheck website is hosted on a Linux server (openSuSE 10.2) with 4GB RAM and two Intel Xeon processors (2.4 GHz). Perl (including BioPerl) scripts are used to parse user input, ARB probematch and UNAfold output, and to create web pages on the fly.
